# Technical aspects in sarcoma surgery – a surgical survey among surgeons at sarcoma centers in Germany and Switzerland

**DOI:** 10.1007/s00423-025-03854-x

**Published:** 2025-09-16

**Authors:** Anne-Christine Zygmunt, Elif Yilmaz, Madelaine Hettler, Michael Ghadimi, Florian Bösch, Jens Jakob

**Affiliations:** 1 Department of General, Visceral, and Pediatric Surgery, University Medical Center Göttingen, Göttingen, Germany; 2 Sarcoma Unit, Department of Surgery, University Medical Center Mannheim, Medical Faculty Mannheim, Heidelberg University, Theodor-Kutzer-Ufer 1-3, 68167 Mannheim, Germany

**Keywords:** Sarcoma surgery, S3 guideline, Survey

## Abstract

**Purpose:**

Surgery is a substantial pillar of extremity sarcoma treatment. There are no standardized, evidence-based strategies to perform a wide R0 resection which is recommended by international and German guidelines. The aim of this study was to use a standardized questionnaire combined with a semi-structured interview to survey the reality of surgical care in German and Swiss sarcoma centers and establish a rationale for future prospective studies to improve postoperative outcome and oncological quality in extremity sarcoma surgery.

**Methods:**

The questionnaire was developed based on a real case of a 32-year-old female patient with a locally advanced soft tissue sarcoma of the proximal thigh. We invited surgeons who were currently treating patients with extremity sarcomas at German and Swiss sarcoma centers.

**Results:**

15 of 24 (62.5%) invited surgeons participated. Participants had a broad type of surgical training and specialization (e.g. general surgery *n* = 5, special visceral surgery *n* = 4, orthopedics and trauma surgery *n* = 8, vascular surgery *n* = 2, plastic surgery *n* = 3). Significant differences (agreement of less than 50%) were found in the planned resection margin at the skin level, the resection planes in other tissues and strategies towards critical structures such as nerves and vessels. Similarities (agreement above 80%) were found in regard to the placement of suction drains and subcutaneous closure.

**Conclusion:**

The current survey shows relevant differences in surgical techniques among sarcoma surgeons at certified sarcoma centers. It is unclear to what extent these differences influence surgical morbidity and oncological outcome. Further studies should be planned to optimize and standardize sarcoma surgery.

**Supplementary Information:**

The online version contains supplementary material available at 10.1007/s00423-025-03854-x.

## Introduction

The rare tumor entity of sarcomas is now increasingly being treated in specialized therapeutic centers. In addition to low incidence, this is also due to the extraordinary heterogeneity of the tumors and their oftentimes persistently poor prognosis [1, 2]. Among others, the German Cancer Society has developed two strategies to improve sarcoma care in Germany. First, the development of an (whenever possible) evidence based guideline for adult soft tissue sarcoma which was published in 2021 [3], which recommends, for example, for lesions larger than 3 cm and suspicious of malignancy to be biopsied, either by core needle biopsy (CNB), or incisional biopsy [4, 5]. Second, the implementation of specialized sarcoma centers which have to prove their structure and process quality in regular audits. The goal of referring all sarcoma patients to a specialized sarcoma board prior to treatment and defining the referral rate as a key quality indicator is one of the key elements of the certification process for German Sarcoma Centers [1]. The rationale behind this being, that coordinated, interdisciplinary treatment is expected to improve disease-free survival in sarcoma patients [2].

Surgical resection of the primary tumor is one of the most important pillars of therapy [6]. Since no surgical specialization covers sarcomas or sarcoma surgery (compare “Facharztkatalog General surgery, Visceral Surgery, Orthopedic and trauma surgery”) or surgical oncology, the surgical approach varies from center to center, largely based on personal experience and surgical traditions. In certified center audits, a lifetime experience of at least 50 sarcoma resections and minimal number of 15 cases per year per sarcoma surgeon is required [7]. The German guideline recommends “wide resection” as the strategy for extremity sarcomas, reflecting the consensus of involved surgical societies [3]. However, a wide variety of technical aspects have not been defined, or examined in direct comparison, so far. Among other things questions posed may refer to the technique of tissue transection, the choice of drainages, the management of lymphatic vessels or the individual oncological radicality. Achieving R0 resection is essential, as tumor-free margins are a key prognostic factor in soft tissue sarcoma [8–10]. Numerous studies have shown that local recurrence rates increase significantly after R1 resection, which radiotherapy or chemotherapy are only partially able to mitigate - especially in biologically aggressive subtypes - thereby underlining the need for an adequately radical surgical approach in these cases [11–14]. In addition to unfavorable resection results, functional limitations, wound healing, or trophic disorders also have a negative impact on the quality of care and might hinder adjuvant treatment.

The individual perioperative surgical steps, including the pre-, the intra- and the postoperative phase, are not systematically defined and examined at international level. The surgical results may diverge greatly and with them the quality of patient care. Knowledge of the perioperative principles applied by various specialists is a prerequisite for joint prospective studies to optimize and standardize sarcoma surgery. Thus, the present study aimed to identify the surgical reality in German and Swiss sarcoma centers using a standardized questionnaire supplemented with a semi-structured interview.

## Materials and methods

**Fig. 1:**
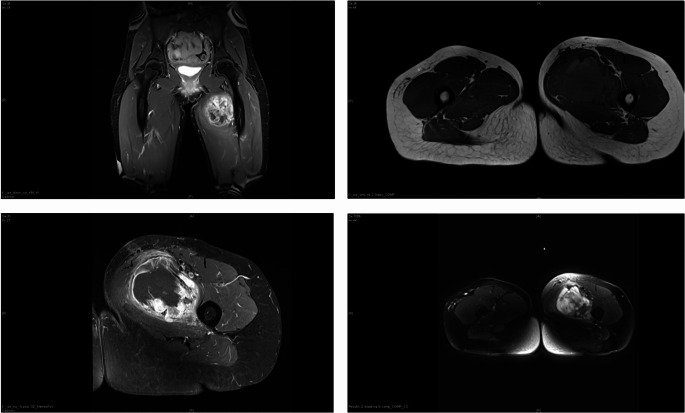
Exemplary selection of included CM-MR (contrast medium-magnetic resonance) images. From top left to bottom right: t1_tse_dixon_cor_KM_W Gadovist, t1_tse_sms tra 2 Steps_COMP, t1_tse_tra_fs post GD_kleinesFoV Gadovist and Results t2 blade tra fs comp_COMP_CO)

The questionnaire was designed based on a real case of a locally advanced soft tissue sarcoma of the proximal thigh in a 32-year-old female patient. It consisted of 20 questions on the pre- (surgical planning, diagnostic standards, handling of previous biopsies), intra- (surgical techniques, material utilization, determination of resection margins) and immediate postoperative (drainage management, wound healing management, rehabilitation) phase (supplemental Table 1). The questionnaire design consisted of open questions, multiple choice questions, and the possibility to add personal comments/explanations. Following consensus with the ethics committee vote, the case vignette and anonymized magnetic resonance images (MRI) (Fig. 1) were provided with the questionnaire. The case vignette was formulated as follows:

*“32-year-old female patient with a non-metastasized*,* undifferentiated pleomorphic sarcoma*,* G3. The diagnosis had been confirmed in advance by incisional biopsy. After 6 cycles of doxorubicin/ifosfamide chemotherapy*,* plus 50 Gy of preoperative radiotherapy. The pre-therapy was very well tolerated. The patient’s general condition and fitness are also very good. The local scars and skin conditions are non-irritating. Please study the MRI images of the findings (see attached DVD) and answer the following questions.”*

The answers were given either in an online survey (https://limesurvey.net) or as a hard copy by fax or e-mail. In September 2022, 24 sarcoma surgeons were invited to take part on a personal basis. The survey was completed in February 2023. The sarcoma-specialized surgeons (major specializations: orthopedics/trauma surgery, general/visceral surgery, plastic surgery) of certified sarcoma centers as well as surgeons with a known focus on sarcoma surgery in Germany and Switzerland were addressed.

The questions were designed as e.g. single choice (yes/no) questions, such as question 2:

*“If a core needle biopsy had been performed*,* would you also excise the canal? Yes or no”.*

Multiple choices were possible for other questions, such as question 6:

*“What do you use to cut the adductor muscles? Monopolar current (needle)*,* monopolar current (knife)*,* bipolar planar sealing (analogous to laparoscopic surgery)*,* bipolar scissors/possibly plus surgical scissors*,* scissors*,* using Overholt clamps with ligature*,* stapler”*,* see appendix.*

In addition, open questions were prepared to allow all possible answers. An answer option “other” was also established for multiple choice questions, which could be answered freely to allow room for improvement and complex answers. Moreover, one question was designed as a figure so that surgeons could mark their individual incision margins.

The questionnaire was then discussed with the participants via telephone conference and again space was provided for additional comments. This allowed all participants to be involved in the concept as well as the evaluation and validation of the questions. As part of the interview section, all questions were asked again to be able to include the existing open questions or additional comments. In addition, the participants were asked to rank the questions by relevance. The additional questions, in particular “*How do you handle deal with the proximity to the vascular-nerve bundle in similar cases?*”, “*How radically do you perform muscle resection in the affected compartment?”*, “*What interval to neoadjuvant radio/chemotherapy do you choose for the time of surgery?*“. “*How do you ensure correct resection margins during surgery?*” and “*How much experience (e.g. cases per year) does the respective responding colleague have?*” were answered as open questions by the interviewees and posed to the subsequent interviewees.

Descriptive statistics were used to analyze and summarize the results. All data were analyzed using Microsoft Excel (Microsoft Corporation, Redmond, VA, USA) and graphs were created using GraphPad Prism (version 8.0, GraphPad Software Inc., Boston, MA, USA). The study was not designed to identify differences in sarcoma surgery between the participating surgeons nor between groups of surgeons trained in different surgical specializations (e.g. general surgery, orthopedic surgery etc.).

## Results

The response rate was 62.5% (*n* = 15/24). The participants had at least one specialist qualification in general surgery (*n* = 5, 33%), with four of them holding additional specializations in visceral (abdominal) surgery and in two cases a third specialization in vascular surgery. One general surgeon held a second specialization in orthopedic surgery and clinically focused on the latter. Six others held primary specializations in orthopedic and trauma surgery (*n* = 6, 40%), of which one held an additional specialization in plastic surgery. Two questionees were primary plastic surgeons (*n* = 2, 13%). Two surgeons were specialized in both visceral and thoracic surgery (*n* = 2; 13%). The number of soft tissue and bone tumor resections performed by the respondents ranged from 25–150/year. In an exploratory analysis of the questionnaire results, we examined whether different results in the answers could be attributed to the specialization of the participating surgeons. We checked whether surgeons from the same specialty always gave the same answers or whether surgeons from different specialties never gave the same answers. This was not the case for any of the questions.

In our survey, all participating surgeons (*n* = 15; 100%) felt that the available imaging contrast medium enhanced MRI (CM-MRI) (Fig. 1) and in general computer tomography (CT) of chest and abdomen was sufficient. Most surgeons (*n* = 11; 73%) stated they would prefer to excise the biopsy incision and would not clip mark the resection cavity after neoadjuvant radiochemotherapy by default (*n* = 12; 80%).

**Fig. 2:**
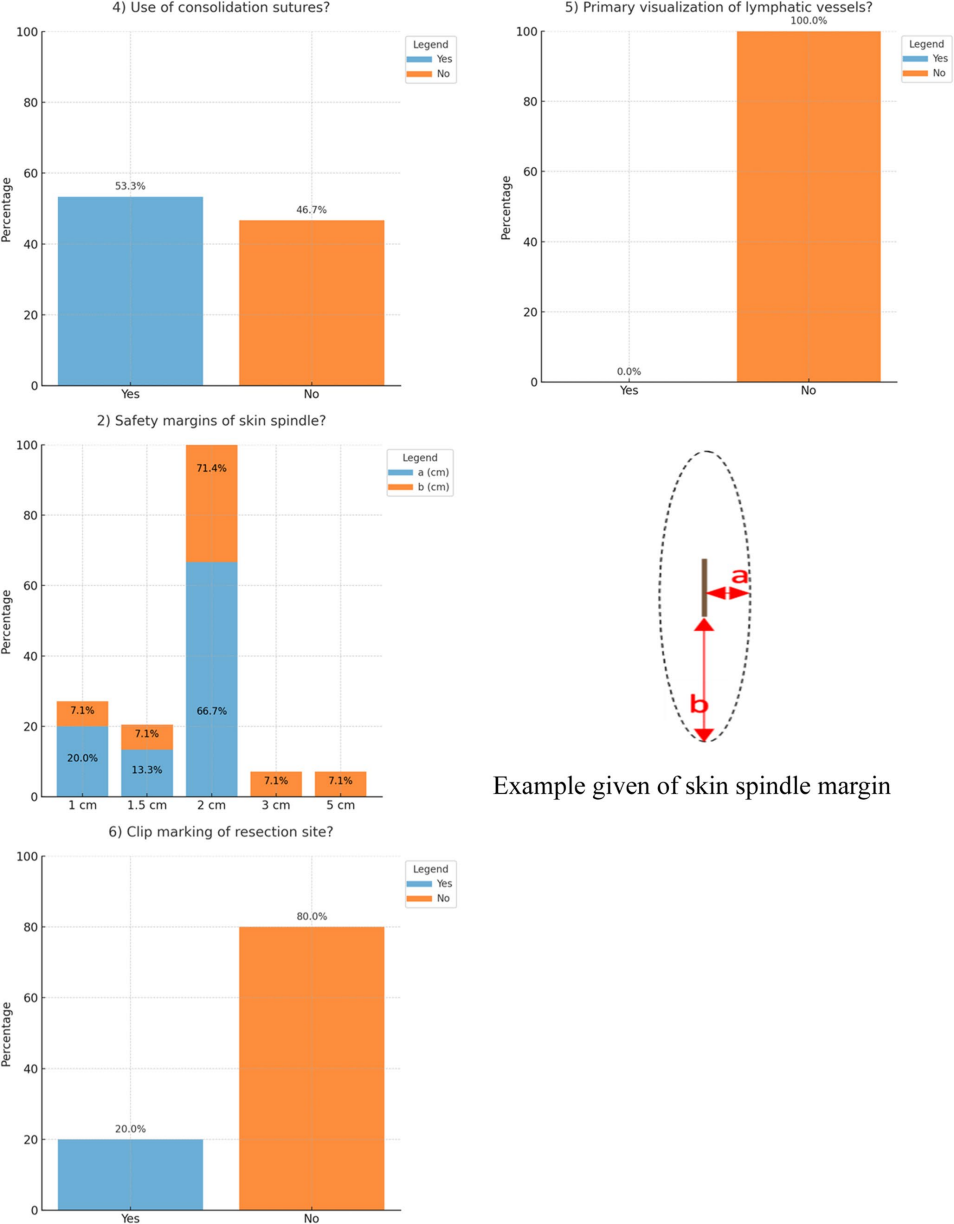
Analysis of Questions 1, 2 (with exemplatory skin spindle and margin provided in questionnaire), 3, 4, 5 and 6. Results presented in percentages

**Fig. 3:**
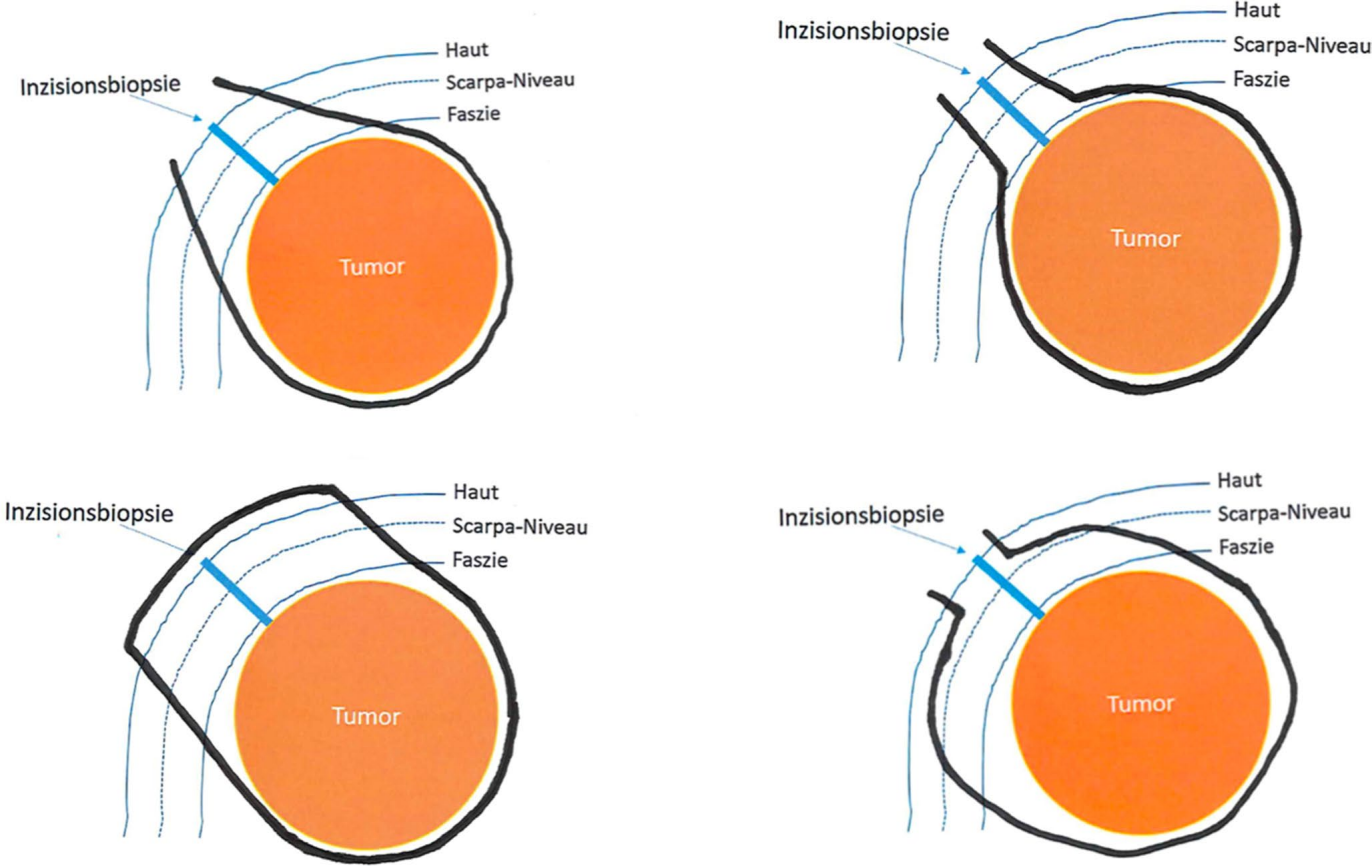
Summary of common resection scopes in response to question 5 “How and where do you cut the subcutaneous tissue and fascia? Please draw in.” From top left to bottom right: pear-shaped (*n* = 6/15), Christmas tree ball-shaped at the level of the muscle fascia (*n* = 6/15), box-shaped (*n* = 2/15) and Christmas tree-shaped at the level of the scarpa fascia (*n* = 1/15) as compared and corrected by way of example in the semi-structured interview

Basic technical principles in (sarcoma) surgery often coincide. In accordance to the figure included in the survey on which participants marked their preferred resection plane, the most frequently chosen safety margin towards the existing incision biopsy was 2 cm horizontally and vertically (*n* = 10; 67%) (see Fig. 2). In high levels of agreement among questionees, the skin was stated to be mainly incised by scalpel (*n* = 14; 93%) and the adductor muscles by monopolar knife (*n* = 10; 67%). Further similarities in sarcoma surgery were also found in the preparation of the tissue. Surgical scissors were preferably used to dissect along the femoral vessels (*n* = 12; 80%). Lymphatic vessels were closed either by ligation (*n* = 10; 67%) and/or clips (*n* = 11; 73%). No surgeon would have the lymphatics primarily imaged (*n* = 15; 100%). Braided (*n* = 10; 67% and *n* = 12; 80%) and absorbable sutures (*n* = 15; 100% and *n* = 15; 100%) were preferably used to adapt the muscle fascia and for subcutaneous suturing. The cutaneous wound was generally Opinions differed widely on the necessity of wound irrigation before closure and which solution should be used, for which most surgeons chose Ringer’s (*n* = 7; 46%). Redon drainages were the most commonly used method for pressure relief (*n* = 11; 73%) and were typically removed based on non-standardized criteria - most often when drainage volume fell below 50 ml/day (*n* = 8; 53%). There was no consensus regarding the appropriate timing for pain-adapted full weight-bearing mobilization. Similarly, no agreement was reached on which instruments were considered essential for the surgical tray. Differences were particularly evident in the access route and wound adaptation. There are no defined standards for approaching the subcutaneous tissue and fascia. Accordingly, the chosen access routes through skin and subcutaneous layers varied significantly among respondents (see Fig. 3). Likewise, only half of the surgeons (*n* = 8; 53%) reported using consolidation sutures to anchor the tumor to the skin or fascia within the resection plane. Across specialties, responses remained heterogeneous, with no discernible pattern suggesting a correlation between surgical discipline and specific answers.

A total of 15 participants took part in the written survey, 10 of whom accepted the opportunity to take part in a follow-up telephone interview. Two of these interviewees were board certified surgeons for general and visceral surgery. One of them estimated performing more than 25 sarcoma resections per year, while no case number was reported for the other. Four participants were orthopedic surgeons, reporting estimated surgical experiences of approximately 140 bone tumors per year, 80–100 tumors per year, nearly 100 soft tissue and bone sarcomas per year, and 120 soft tissue tumors annually, respectively, of which around 60 were personally performed. Two interviewees were specialized in plastic surgery, with one reporting approximately 60 sarcoma operations annually within their field, and the other indicating experience with around 150 soft tissue and bone tumor resections per year. Two additional interviewees held combined specialties: one in general and orthopedic surgery, with approximately 70 sarcoma resections annually, and one in visceral and thoracic surgery, estimating 80–100 resections per year. With regard to the additional questions posed by the interviewees themselves, the questions “*How do you handle tumors with proximity to the vascular-nerve bundle (or other critical structures*,* e.g. periosteum) in similar cases?“*, “*How radically do you perform muscle resection in the affected compartment?*“, “*What interval to neoadjuvant radio/chemotherapy is chosen for the time of surgery?*” and “*How do you ensure correct margins during surgery?”* but also *“How much experience (e.g. cases per year) does the respective responding colleague have?*” were of greatest interest for the majority of surgeons.

The question of resection margins near critical structures, such as the neurovascular bundle, revealed notable differences in surgical approach. Overall, the interviewees agreed that vascular preservation was possible and should be aimed for in the presented case. However, opinions diverged when surgeons were asked to consider hypothetical scenarios involving closer proximity of the tumor to vascular and/or neural structures. Some favored a radical R0 resection approach, particularly in younger patients, if necessary even including vascular replacement. Others argued in favor of preserving critical structures and accepting an R1 resection, provided that adjuvant radiotherapy would be administered. It was also emphasized that a macroscopic and microscopic R1 resection differ significantly in terms of oncological relevance. In borderline cases, structures such as the adventitia or epineurium should be resected to provide oncological safety.

Another relevant question for the participants was the radicality or extent of the muscle resection in the affected compartment. Surgeons agreed that there should be a surrounding layer of healthy tissue, as called for in the guidelines. For example, anatomical barriers such as fascia were considered acceptable resection borders, provided there was adequate mobility and separation from surrounding healthy tissue. However, the question arose regarding the appropriate longitudinal extent of muscle or muscle group resection. Even though different therapeutic approaches were also discussed here, all stated that the aim should be to preserve function, especially in patients of a young age, but not to the disadvantage of the oncological outcome. Particular emphasis was placed on the tumor’s biological behavior. Only two participants gave a concrete answer regarding the proximal/distal distance of the resection area to the tumor, stating 1–2 cm.

Regarding the general question of how to ensure sufficient intraoperative resection margins and spatial orientation within the tissue, most interviewees emphasized the importance of maintaining a healthy displacement layer. They reported typically using their own hand to stabilize the tumor during dissection, thereby creating an estimated one-finger-thick safety margin on all sides. This approach is preceded by a thorough review of preoperative imaging and mental mapping of relevant anatomical landmarks. Only one surgeon reported using pre- and intraoperative ultrasound as well as tape measurements to support intraoperative orientation.

There was agreement on the timing of surgery after previous therapy. Usually, it was at around four to six weeks after conclusion of neoadjuvant treatment. Two of the interviewees stated that they preferred adjuvant radiotherapy to preoperative radiotherapy due to the increased risk of wound healing disorders and the persistently complex nature of the resection.

Additional topics considered relevant included the preferred sequence of multimodal therapy, plastic reconstruction techniques, compression strategies, and the management of complications - particularly seroma treatment. Likewise, more detailed postoperative rehabilitation protocols and complex therapeutic approaches, such as indications for isolated limb perfusion. Although these aspects were mentioned, they were not further explored by the participants.

## Discussion

More than half of all sarcoma patients present without metastases at the time of initial diagnosis [15–18]. For this group, wide R0 resection is the most important treatment option - not only from an oncological perspective, but also in terms of quality of life [19]. The surgical approach should be embedded in a multimodal treatment plan and discussed by a dedicated multidisciplinary sarcoma board [2]. It is noteworthy that comparative studies on surgical techniques in sarcoma treatment remain scarce, regardless of whether they focus on instrumentation, operative methodology, or strategic approaches. To evaluate the current situation and address this gap, we conducted a survey among sarcoma centers in German-speaking countries.

The key challenges in extremity sarcoma surgery are achieving tumor-free margins, preserving function, and avoiding postoperative complications. Achieving these objectives can be challenging due to the lack of clearly defined and standardized surgical protocols. It is therefore not surprising that the survey identified the definition of resection margins and the management of critical structures - such as blood vessels and nerves - as the main areas of divergence, as these are also subjects of ongoing and controverse debate in the literature [20–22].

”Wide resection” as a surgical standard for the treatment of adult soft tissue sarcomas has been known since the 1980 s and at that time demonstrated its equivalence to amputation-based surgery [10]. In the meantime, it has also found its way into the German S3 guideline “Adult soft tissue sarcomas” as the gold standard for extremity sarcomas [19]. Clear surgical margins are of particular relevance here, given their established association with reduced local recurrence and improved survival - especially in liposarcoma and high-grade soft tissue sarcoma [23, 24]. But uncertainties remain, especially in clinical practice: how wide is wide? How to ensure wide enough margins?

In contrast, there are clear recommendations for early-stage resection in the case of malignant melanoma [25]. Based on current evidence, surgical guidelines for melanoma have been revised and uniformly established. The heterogeneity of sarcoma subtypes, however, each with distinct biological behavior and aggressiveness, complicates the development of standardized surgical guidelines. As already summarized by Kawaguchi et al., fascia, visceral or parietal sheaths, bone skins and other barriers are considered tumor-arresting and thus adequate resection layers [26]. Specific margin widths for “wide resection” - based on factors such as preoperative therapy, tumor grading, and anatomical location - have been discussed in the literature. However, none of these proposals have been incorporated into current guidelines or established clinical practice. Consequently, both surgical results and oncological outcomes are inconsistent, with some extensive resections causing substantial functional impairments in the preserved tissue.

The present study highlights that surgical approaches differ considerably among experts. Only a few respondents reported using intraoperative sonography or tape measurements to determine resection margins from the palpable tumor capsule. Most surgeons instead relied on manual palpation, using the thickness of their fingers as a reference for an adequate safety margin. When asked to illustrate their individual resection planes around the tumor capsule, participants demonstrated differing strategies regarding both incision lines and dissection planes.

Our survey also revealed a great need for further discussion of the management of neurovascular bundles in close proximity to the tumor, particularly in cases of suspected or direct contact. As this issue was repeatedly raised during the interviews, it was then incorporated into the questionnaire as an open-ended, additional question posed to all subsequent interview partners. Overall, interviewees agreed that, in the case vignette presented here, vascular structures should generally be preserved.

However, the strong interest in this topic and the range of differing opinions once again highlighted the need for structured investigation into its clinical implications. Several participants argued in favor of a patient’s young age, calling for a more radical approach, including vessel replacement if adventitial dissection proved insufficient to achieve R0 resection. Others favored preserving critical structures, emphasizing the role of interdisciplinary perioperative treatment strategies, especially radiotherapy, as a means to compensate for a potentially closer resection margin.

In 2014, in retrospectively analyzed data from over 1000 patients treated between 1989 and 2009, O’Donnell et al. showed that the 5-year local recurrence-free survival rate was only marginally improved in patients who underwent R0 resection of critical structures (e.g. vessels, nerves, or bone) with or without reconstruction, compared to those in whom microscopic positive margins at these sites were accepted in the context of multidisciplinary treatment [27]. The study further showed that positive margins in the tumor bed or other unintended locations were associated with significantly lower local control rates than positive margins at anatomically critical sites. In addition, different histological subtypes exhibit variable growth and infiltration patterns, occasionally resulting in unexpected positive margins in such unfavorable areas - for example, in cases of undifferentiated pleomorphic sarcoma.

Apart from the debated issue of how to manage critical structures, the survey also revealed several areas of consensus. There was a consensus on maintaining a recovery period of 4–6 weeks between neoadjuvant therapy and surgery, excision of the biopsy incision, closure of lymphatic vessels as well as on general wound management drainage usage.

In our survey, 73% of respondents stated that they would resect the core needle biopsy (CNB) tract en bloc, if identifiable. This practice aligns with current guideline recommendations, which strongly advocate for en bloc resection of especially incisional but also CNB tracts [4]. The rationale for this approach - preventing potential tumor cell seeding and thus subsequent local recurrence – appears largely intuitive [28]. Local recurrences that can be clearly attributed to the (core needle) biopsy, as well as microscopic evidence of tumor cells in the biopsy tract after resection, are rare [29]. In the available report, there was no relevant difference in the behavior of local recurrences or worsened prognosis after tract resection compared to non-resection. Furthermore, as expected, a better oncological course was recorded in patients with adequate perioperative multidisciplinary therapy, regardless of tract resection. Thus, the benefit clearly outweighs the risk, even in high-grade tumors. Similar data is also available for gastrointestinal stromal tumors (GIST): GISTs - for which perforation or R2 resection is associated with a strikingly poor local recurrence rate and prognosis - also show no clear evidence of worsened oncological outcomes following biopsy.

When resecting large tumors such as sarcomas, poses a significant challenge to postoperative healing. Previous studies have shown that 49% of patients experience complications following extremity sarcoma resection, with wound infection (23.3%) and dehiscence (19.5%) being particularly common; in 13.2% of cases, surgical revision was required [30–32]. In addition to smoking, pre-existing cardiovascular disease and elevated BMI, the localization of the sarcoma on the lower extremity, prolonged operative time, plastic reconstruction and neoadjuvant radiotherapy were identified as risk factors [33, 34]. In our study, there was general consensus on irrigation of the wound prior to wound closure. Interestingly, there were differences in wound closure techniques. Only about half of the surgeons reported using non-absorbable single button or a staple suture, indicating a lack of uniform practice - particularly relevant given the frequent occurrence of wound dehiscence. The use of adjunctive materials such as epicutaneous vacuum-assisted closure systems was rare, with only two surgeons indicating regular use. Yet, evidence suggests that such systems may help reduce wound complications in patients undergoing preoperative radiotherapy for lower extremity sarcoma [35].

Which resection technique and extent, which instruments are used and how, how wound closure is carried out, but also how drainages are positioned and when they are removed, have not yet been systematically correlated with wound complications, as there are no structured studies on this. In a first analytical step, we asked about the materials and techniques employed by surgeons to identify practice variations and explore their potential implications. The findings revealed that the selection of tools and surgical strategies is closely linked to the surgeon’s background and specialty-specific training. Although the German Cancer Society’s certification process requires demonstrated surgical experience, it is not confined to a particular specialty. Our respondent group included surgeons from six distinct medical disciplines. Due to differences in professional training, the areas of expertise and choice of surgical techniques among certified sarcoma surgeons can vary. A simple example is the soft tissue preparation technique. A visceral surgeon, for instance, is accustomed to using sealing instruments (e.g. Ligasure™) during abdominal surgeries and prefers to use these instruments when performing resections of extremity tumors as well. Conversely, orthopedic and trauma surgeons are generally trained in clamp-and-ligation techniques for hemostasis, as commonly used in fracture surgery, and are therefore less inclined to employ vessel sealing devices in sarcoma resections. This illustrates how specialty-specific training influences intraoperative decision-making. Technical aspects like muscle transection and hemostasis are highly relevant, especially considering postoperative complication rates exceeding 15%, which are associated with increased healthcare costs and may delay or impair adjuvant treatment, ultimately affecting oncological results [31, 32]. Cross-disciplinary exchange therefore may offer valuable opportunities: technical nuances and methods can be shared and adapted to improve surgical outcomes [36]. Technical aspects like muscle transection and hemostasis remain highly relevant, especially considering postoperative complication rates exceeding 15%, which are associated with increased healthcare costs and may delay or impair adjuvant treatment, ultimately affecting oncological results.

In an exploratory analysis of the questionnaire results, we examined whether different results in the answers could be attributed to the specialization of the participating surgeons. We checked whether surgeons from the same specialty always gave the same answers or whether surgeons from different specialties never gave the same answers. This was not the case for any of the questions.

This study has several limitations. It focuses on a single standardized case vignette rather than a broader set of clinical scenarios, which may limit the generalizability of its findings. The number of participants was relatively small, and the sample may not be fully representative of the wider community of surgeons treating sarcoma. Moreover, the study was not designed or powered to detect statistically significant differences between surgical specialties.

Nevertheless, the purpose of this exploratory survey was not to generate conclusive comparisons, but rather to identify general trends and divergences in surgical decision-making; particularly in a field where no specialty currently defines formal training requirements or minimum caseloads for soft tissue sarcoma surgery. As an alternative, a series of clinical cases could have been presented and distributed in the format of a ring trial, following the model already established for multimodal therapy decision-making within certified German sarcoma centers [38]. However, such a procedure would have been very complex in terms of the individual techniques involved. The current approach is sufficient for exploratory analysis and we will be able to select single diverging aspects to be evaluated in prospective studies or a ring trial.

In conclusion, the current survey shows clear differences in the surgical approach of a “wide R0-resection”. Studies focusing not only on technical procedures but also examining their possible correlations with morbidity outcomes, such as wound healing, should be developed. Future research may also focus more explicitly on differences between specialties. This foundation may allow sarcoma surgeons to systematically define key procedural steps in extremity sarcoma surgery, laying the groundwork for the rational design of surgical trials. However, we believe that establishing cross-specialty educational strategies - regardless of surgical background - is of even greater importance to improve surgical care for sarcoma patients. In Germany, certified sarcoma centers, CHIR-Net, and patient advocacy groups provide a strong collaborative framework to drive such research efforts forward [37, 38].

## Supplementary Information

Below is the link to the electronic supplementary material.

ESM 1 (DOCX 33.4 KB)

## Data Availability

No datasets were generated or analysed during the current study.

## Abbreviations

BMI: Body mass index
CM: Contrast medium
CNB: Core needle biopsy
CT: Computer tomography
G3: Grading, poorly differentiated carcinoma
GIST: Gastrointestinal stromal tumor
HR: Hazard ratio
MR: Magnetic resonance
MRI: Magnetic resonance imaging
R: Resection margin
