# Supplementary material for: Technical aspects in sarcoma surgery – a surgical survey among surgeons at sarcoma centers in Germany and Switzerland
Source: Langenbecks Arch Surg. 2025 Sep 16;410(1):272. doi: 10.1007/s00423-025-03854-x (PMC12441070; doi:10.1007/s00423-025-03854-x)
Supplement: Supplementary file 1 — (DOCX 33.4 KB) [file 423_2025_3854_MOESM1_ESM.docx]

**Supplementary**

| Survey questions.  Results presented in absolute numbers and percentages in parenthesis, ≥80 %marked as ‘consent’, <50% marked as ‘no consent’. | |  | |  |  |
| --- | --- | --- | --- | --- | --- |
| 1 | What is your specialty? | (Individual answer) | |  |  |
| 2 | Do you consider the available imaging (CM-MRI, CT) sufficient to plan the surgery? | Yes | | 15/15 (100) | consent |
|  |  | No | | 0/15 (0) | no consent |
| 3 | If a core needle biopsy had been performed, would you also excise the canal? | Yes | | 11/15 (73) | no consent |
|  |  | No | | 4/15 (26,7) | no consent |
| 4 | Which instrument do you use for skin incision? | Scalpel | | 14/15 (93,3) | consent |
|  |  | Monopolar cautery (needle) | | 1/15 (6,7) | consent |
| 5 | How and where do you cut the subcutaneous tissue and fascia? | (Figure) | | |  |
| 6 | Do you utilise consolidation sutures to fix the tumor to the skin or fascia in the resection plane? | Yes | | 8/15 (53,3) | no consent |
|  |  | No | | 7/15 46,7) | no consent |
| 7 | What do you use to dissect the adductor muscles? | Monopolar cautery (needle) | | 4/15 (26,7) | no consent |
|  |  | Monopolar cautery (knife) | | 10/15 (66,7) | no consent |
|  |  | Bipolar planar sealing (analogous to laparoscopic surgery), | | 1/15 (6,7) | no consent |
|  |  | Bipolar scissors/possibly plus surgical scissors | | 5/15 (33,3) | no consent |
|  |  | Scissors | | 0/15 (0) | no consent |
|  |  | Overholt clamps with ligature | | 0/15 (0) | no consent |
|  |  | Stapler | | 1/15 (6,7) | no consent |
| 8 | What do you use to dissect along the femoral vessels? | Bipolar scissors | 1/15 (6,7) | | no consent |
|  |  | Scissors | 12/15 (80) | | consent |
|  |  | With magnifying glasses | 6/15 (40) | | no consent |
|  |  | Without magnifying glasses | 7/15 (46,7) | | no consent |
|  |  | Overholt with monopolar current | 1/15 (6,7) | | no consent |
| 9 | How do you seal lymphatic vessels? | Ligation | 10/15 (66,7) | | no consent |
|  |  | Clips | 11/15 (73,3) | | no consent |
|  |  | Bipolar planar sealing | 1/15 (6,7) | | no consent |
|  |  | Monopolar cautery | 2/15 (13,3) | | no consent |
|  |  | Bipolar forceps cautery | 3/15 (20) | | no consent |
|  |  | Transfixation suture ligature | 1/15 (6,7) | | no consent |
|  |  | No answer | 1/15 (6,7) | | no consent |
| 10 | Do you primarily visualize lymphatic vessels? | Yes | 0/15 (0) | | no consent |
|  |  | No | 15/15 (100) | | consent |
| 11 | Do you perform standardized clip marking of the resection site after radiochemotherapy? | Yes | 3/15 (20) | | no consent |
|  |  | No | 12/15 (80) | | no consent |
| 12 | Which solution do you use to irrigate a wound before closure? | Aqua | 3/15 (20) | | no consent |
|  |  | Ringer’s solution | 7/15 (46,7) | | no consent |
|  |  | H_2_O_2_ | 3/15 (20) | | no consent |
|  |  | Antiseptic solution (Lavasept, Granudacyn, ..) | 1/15 (6,7) | | no consent |
|  |  | Natriumchlorid | 3/15 (20) | | no consent |
|  |  | None | 1/15 (6,7) | | no consent |
| 13 | If the muscle fascia can be adapted without creating major cavity, how do you adapt the muscle fascia? Which suture material and which suturing technique do you choose? | Monofilament | 4/15 (26,7) | | no consent |
|  |  | Braided | 10/15 (66,7) | | no consent |
|  |  | Absorbable | 15/15 (100) | | consent |
|  |  | Non-absorbable | 0/15 (0) | | no consent |
|  |  | Continuous suture | 5/15 (33.3) | | no consent |
|  |  | Single button | 11/15 (73,3) | | no consent |
| 14 | Which suture material and which suturing technique do you choose for subcutaneous suturing? | Monofilament | 2/15 (13,3) | | no consent |
|  |  | Braided | 12/15 (80) | | consent |
|  |  | Absorbable | 15/15 (100) | | consent |
|  |  | Non-absorbable | 0/15 (0) | | no consent |
|  |  | Continuous suture | 0/15 (0) | | no consent |
|  |  | Single button | 13/15 (86,7) | | consent |
|  |  | Grasping of skin | 7/15 (46,7) | | no consent |
|  |  | No grasping of skin | 7/15 (46,7) | | no consent |
| 15 | Which suturing technique do you choose for cutaneous suturing? | Single button, non-absorbable | 7/15 (46,7) | | no consent |
|  |  | Intracutaneous suture, absorbable | 3/15 (20) | | no consent |
|  |  | Intracutaneous suture, non-absorbable | 1/15 (6,7) | | no consent |
|  |  | Staple suture | 6/15 (40) | | no consent |
|  |  | Epicutaneous VAC-therapy on top of incision | 1/15 (6,7) | | no consent |
| 16 | Where do you place the drainage? | Figure |  | |  |
| 17 | Which drainage do you use? | Redon-Drainage | 11/15 (73,3) | | no consent |
|  |  | Easyflow-Drainage | 1/15 (6,7) | | no consent |
|  |  | Blakes-Drainge | 1/15 (6,7) | | no consent |
|  |  | Jackson-Pratt | 1/15 (6,7) | | no consent |
|  |  | Robinson-Drainage | 1/15 (6,7) | | no consent |
| 18 | Which criteria do you apply for removal of drainages? (Open question) | Secretion < 30ml/24h | 1/15 (6,7) | | no consent |
|  |  | Secretion < 40 ml/24h | 2/15 (13,3) | | no consent |
|  |  | Secretion < 50ml/24h | 8/15 (53,3) | | no consent |
|  |  | Secretion < 100ml/24h | 4/15 (26,7) | | no consent |
|  |  | Time < 48 h | 1/15 (6,7) | | no consent |
|  |  | Time ≥ 3 days | 2/15 (13,3) | | no consent |
|  |  | Time < 7 days | 1/15 (6,7) | | no consent |
|  |  | Time 7 – 10 days | 1/15 (6,7) | | no consent |
|  |  | Time < 14 days | 1/15 (6,7) | | no consent |
| 19 | When can pain-adapted full weight-bearing physical activity be performed? (Open question) | As soon as possible | 6/15 (40) | | no consent |
|  |  | Individually | 1/15 (6,7) | | no consent |
|  |  | After 1 day bed rest | 4/15 (26,7) | | no consent |
|  |  | After 2 days bed rest | 2/15 (13,3) | | no consent |
|  |  | After 3 days bed rest | 1/15 (6,7) | | no consent |
|  |  | After 5 days bed rest | 1/15 (6,7) | | no consent |
|  |  | After 2 weeks (without radiation) | 1/15 (6,7) | | no consent |
|  |  | After 4 weeks postoperative, until then 20 kg | 1/15 (6,7) | | no consent |
|  |  | After radiation, after 4 weeks postoperative full-weight. Until then half-weight | 1/15 (6,7) | | no consent |
| 20 | Which instruments should not be missing in surgery? (Open question) | Scissors/Overholt/Forceps | 1/15 (6,7) | | no consent |
|  |  | Basic surgical tray | 1/15 (6,7) | | no consent |
|  |  | Standard | 1/15 (6,7) | | no consent |
|  |  | Clips | 2/15 (13,3) | | no consent |
|  |  | Retractors/hooks, anatomical dissection forceps, electrical cautery, surgical tape measure |  | |  |
|  |  | Dissection scissors | 4/15 (26,7) | | no consent |
|  |  | Overholt | 2/15 (13,3) | | no consent |
|  |  | Ligasure | 2/15 (13,3) | | no consent |
